# Revealing Surfactant Effect of Trifluoromethylbenzene in Medium‐Concentrated PC Electrolyte for Advanced Lithium‐Ion Batteries

**DOI:** 10.1002/advs.202206648

**Published:** 2023-02-19

**Authors:** Mingsheng Qin, Ziqi Zeng, Xiaowei Liu, Yuanke Wu, Renjie He, Wei Zhong, Shijie Cheng, Jia Xie

**Affiliations:** ^1^ State Key Laboratory of Advanced Electromagnetic Engineering and Technology School of Electrical and Electronic Engineering Huazhong University of Science and Technology Wuhan Hubei 430074 P. R. China; ^2^ State Key Laboratory of Materials Processing and Die & Mould Technology School of Materials Science and Engineering Huazhong University of Science and Technology Wuhan Hubei 430074 P. R. China; ^3^ State Key Laboratory of Advanced Technology for Materials Synthesis and Processing Wuhan University of Technology Wuhan Hubei 430070 P. R. China

**Keywords:** adsorption, electrolytes, lithium‐ion batteries, solid electrolyte interface, solvation structure

## Abstract

Despite wide‐temperature tolerance and high‐voltage compatibility, employing propylene carbonate (PC) as electrolyte in lithium‐ion batteries (LIBs) is hampered by solvent co‐intercalation and graphite exfoliation due to incompetent solvent‐derived solid electrolyte interphase (SEI). Herein, trifluoromethylbenzene (PhCF_3_), featuring both specific adsorption and anion attraction, is utilized to regulate the interfacial behaviors and construct anion‐induced SEI at low Li salts’ concentration (<1 m). The adsorbed PhCF_3_, showing surfactant effect on graphite surface, induces preferential accumulation and facilitated decomposition of bis(fluorosulfonyl)imide anions (FSI^−^) based on the adsorption–attraction–reduction mechanism. As a result, PhCF_3_ successfully ameliorates graphite exfoliation‐induced cell failure in PC‐based electrolyte and enables the practical operation of NCM613/graphite pouch cell with high reversibility at 4.35 V (96% capacity retention over 300 cycles at 0.5 C). This work constructs stable anion‐derived SEI at low concentration of Li salt by regulating anions–co‐solvents interaction and electrode/electrolyte interfacial chemistries.

## Graphical abstract

Trifluoromethylbenzene (PhCF_3_), as surfactant, is introduced into propylene carbonate (PC)‐based electrolyte to regulate interfacial behaviors in lithium‐ion batteries. The adsorbed PhCF_3_ on graphite surface facilitates bis(fluorosulfonyl)imide anions’ (FSI^−^) accumulation and decomposition at graphite/electrolyte interface through the adsorption–attraction–reduction mechanism. As a result, the graphite is kinetically stabilized in medium‐concentrated PC electrolyte with high reversibility.

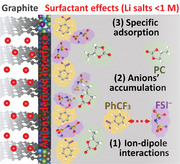

## 1 Introduction

Despite the nearly doubled energy density and widespread utilization of lithium‐ion batteries (LIBs) among portable electronics and grid‐scale energy storage over the past several decades, carbonaceous materials, primarily graphite, are still dominant anode in commercial LIBs.^[^ ^1^, ^2^, ^3^ ^]^ Moreover, the intercalation chemistry of graphite within LIBs relies heavily on ethylene carbonate (EC) solvent for kinetically passivating graphite surface by forming robust SEI.^[^ ^4^, ^5^ ^]^ As a result, EC has established the cardinal roles in commercial electrolytes for LIBs. Nevertheless, the high melting point (36.4 °C) and detrimental electrode cross‐talk in EC‐based electrolyte stimulate new impetus to develop EC free electrolytes.^[^ ^6^, ^7^ ^]^ For instance, propylene carbonate (PC), EC's close sibling, has been rejuvenated as a promising alternative due to its wide liquid range (−48.8 to 242 °C) and high dielectric constant in recent years.^[^ ^8^, ^9^ ^]^ However, the employment of PC as single‐solvent electrolyte proves infeasible because of solvent co‐intercalation and endless decomposition, leading to detrimental graphite exfoliation and cell failure.^[^ ^10^, ^11^ ^]^

It is believed that the electrochemical behavior of graphite is intimately linked to Li^+^ solvation chemistry and dictated by the formed SEI in LIBs, which inspires two avenues to enable graphite electrode with PC‐dominating electrolyte, namely regulating solvation structure and constructing robust interphase.^[^ ^12^, ^13^, ^14^, ^15^, ^16^ ^]^ Indeed, some novel additives, such as Li salts with tailor‐designed structure and new film‐forming molecules, have been tentatively added into PC‐based electrolytes to adjust SEI composition and successfully guaranteed reversible cycling of graphite.^[^ ^17^, ^18^ ^]^ Recently, the super‐concentrated electrolytes (SCEs) and diluted SCEs are designed to construct anion‐dominated coordination. More anions tend to appear on graphite surface in SCEs and decompose to generate a compact inorganic‐rich SEI.^[^ ^19^, ^20^, ^21^ ^]^ However, the inherent high viscosity and high cost for SCEs fail to meet the practical demands. Noticeably, metal coating and surface modification are widely employed on graphite or Li metal surface for enhancing electrochemical performance,^[^ ^22^, ^23^, ^24^ ^]^ which inspires the idea of employing surfactant into PC electrolyte to guide interfacial behaviors so that reliable SEI and superior solvation chemistry can be simultaneously obtained at relatively low Li salts concentration. For instance, ethyl isothiocyanate are added into PC electrolyte since its preferential adsorption and decomposition.^[^ ^17^ ^]^ Ammonium perfluoro(2‐methyl‐3‐oxahexanoate) is used as surfactant to modify the electric double layer at Li metal interfaces.^[^ ^25^ ^]^ In addition, rhodamine B^[^ ^26^ ^]^ and 1,3,6‐hexanetricar‐bonitrile^[^ ^27^ ^]^ are also used to regulate interfacial chemistry in LIBs and LMBs respectively.

**Figure 1:**
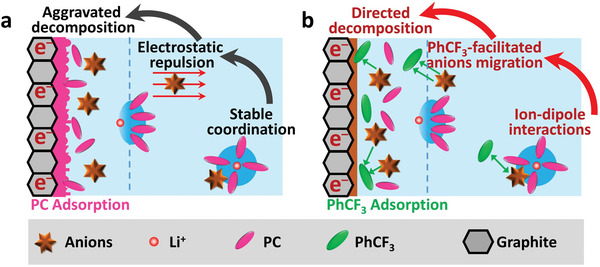
The interfacial behaviors of Li^+^‐PC complexes and anions near the surface of graphite electrode in a) blank and b) PhCF_3_‐containing electrolyte.

Herein, trifluoromethylbenzene (PhCF_3_), an aprotic co‐solvent featuring electron‐withdrawing group (–CF_3_) and benzene ring, is proposed to regulate the interfacial behaviors of anions near graphite surface via the ion–dipole interactions. Differing from common additives (e.g., fluoroethylene carbonate (FEC) and vinylene carbonate (VC)), which participate in Li^+^ solvation and self‐sacrificially decompose on graphite surface to construct additive‐derived SEI, PhCF_3_ stays out of Li^+^ coordination and preferentially adsorbs on graphite surface, facilitating anions’ decomposition and inhibiting PC co‐intercalation. The detailed working mechanism is illustrated in **Figure** **1**. In the blank electrolyte, anions depart from Li^+^ solvation shell once approaching graphite due to the long‐range electrostatic force during cathodic process (Figure 1a), resulting in accumulation of Li^+^‐PC complexes near graphite surface.^[^ ^28^, ^29^, ^30^ ^]^ The dominated decomposition of PC and resulting fluffy SEI are responsible for the following graphite exfoliation. When preferential adsorption of PhCF_3_ proceeds on graphite surface due to *π*–*π* stacking, the ion–dipole interaction between anion and PhCF_3_ compensates electrostatic repulsion and promotes anions’ accumulation on graphite surface (Figure 1b). The robust SEI, derived from anions’ decomposition, stabilizes graphite anode in PC electrolyte even at low concentration of Li salts (<1 m). As a result, PhCF_3_ circumvents the weakness of SCEs (high viscosity and cost) and realizes stable interface simultaneously. Thus, PhCF_3_‐regulated SEI enables reliable operation of NCM613/graphite pouch cell over 300 cycles with 96% capacity retention. Moreover, such electrolyte also inherits wide liquid range (−70 to 160 °C) and high‐voltage compatibility (4.4 V for NCM811/Li cells, 4.35 V for NCM613/graphite pouch cell).

## 2 Results and Discussions

### 2.1 PhCF_3_–Anions Interaction and Solvation Structure

**Figure 2:**
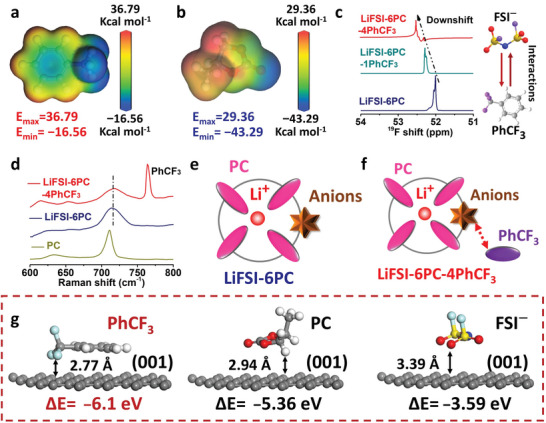
PhCF_3_–anions interactions and specific adsorption. The electrostatic potential (ESP) results of a) PhCF_3_ and b) PC. c) NMR results of FSI^−^ and d) Raman results of PC in different electrolytes. The illustrations for the solvation structure of e) LiFSI‐6PC and f) LiFSI‐6PC‐4PhCF_3_. g) Adsorption energies of different species on graphite surface.

The electrostatic potential (ESP) calculations are performed to investigate the ion–dipole interactions between PhCF_3_ and FSI^−^ anion. As shown in **Figure** **2**a, the strong electron‐withdrawing effect of fluorine leads to electron accumulation on trifluoromethyl, showing *E*_max_ of 36.79 and *E*_min_ of −16.56 kcal mol^−1^. The high *E*_max_ value of PhCF_3_ indicates high thermodynamic possibility of interaction with anion.^[^ ^31^, ^32^ ^]^ Moreover, the counterpart of trifluoromethyl in PhCF_3_, as a Lewis acid center, can strongly attract the negative moiety of FSI^−^ anions.^[^ ^33^ ^]^ The interactions between PhCF_3_ and anions are further revealed by ^19^F NMR (LiFSI:PC molar ratio of 1:6 is denoted as L6PC, LiFSI:PC:PhCF_3_ molar ratio of 1:6:4 is denoted as L6PC4Ph), showing successive downshift from 52.1 to 52.5 ppm for FSI^−^ after adding PhCF_3_ (Figure 2c). Accordingly, the consistent ^19^F upshift is also observed in PhCF_3_ (Figure S1, Supporting Information), demonstrating the ion‐dipole interaction between PhCF_3_ and FSI^−^. As a result, such interaction might facilitate the dissolution of Li salts in PC, leading to intensified Li^+^‐PC strength as denoted by the downshift of ^1^H peak for PC (Figure S2, Supporting Information).

Raman spectra are compared to investigate electrolyte structure (Figure S3, Supporting Information). Blue shift is observed for PC after LiFSI dissolution (from PC to LiFSI‐6PC), indicating the formation of Li^+^‐PC complexes (Figure 2d).^[^ ^34^ ^]^ After adding PhCF_3_, the Raman peak keeps original position and shape, implying the preserved solvation structure (Figure 2d and Figure S4, Supporting Information). It is reasonable to get the preserved coordination chemistry after adding PhCF_3_. The value of *E*_min_ for PhCF_3_ (−16.56 kcal mol^−1^) is much higher than that of PC (−43.29 kcal mol^−1^) (Figure 2a,b), indicating the inferior coordinating capability of PhCF_3_ to Li^+^ compared to that of PC, agreeing well with the negligible dissolution of Li salts in PhCF_3_ solvent (Figure S5, Supporting Information). As a result, the LiFSI‐6PC shows PC‐dominated primary solvation shell with anions situated within the outer solvation shell (Figure 2e). After introducing PhCF_3_ (L6PC4Ph), the primary Li^+^ solvation structure keeps intact while FSI^−^ is attracted by PhCF_3_ molecules (Figure 2f).

DFT calculations are applied to investigate the preferential adsorption of PhCF_3_. Figure 2g exhibits the optimized geometrical configurations of PhCF_3_, PC, and FSI^−^ on graphite surface. The shortest distance between PhCF_3_ and graphite is 2.77 Å, smaller than that of PC (2.94 Å) and FSI^−^ (3.39 Å), indicating the superior engagement of PhCF_3_ on graphite because of an inherent electrostatic advantage (*π*–*π* stacking mode). The calculated adsorption energies for PhCF_3_, PC, and FSI^−^ on graphite (001) plane are −6.1, −5.34 and −3.59 eV, respectively, further indicating the energetic superiority of PhCF_3_ on graphite surface. As demonstrated by the diagram of charge density difference (Figure S6, Supporting Information), the charge distributes uniformly over benzene ring with PhCF_3_ adsorption, while the charge induced by PC on graphite accumulates mainly on two adjacent surface C atoms. The larger interaction cross section for PhCF_3_ indicates its overwhelming adsorption on graphite surface.^[^ ^17^ ^]^ Thus, PhCF_3_ exhibits two characteristics: 1), the positive center over PhCF_3_ attracts FSI^−^ via ion–dipole interactions without altering Li^+^ solvation structure; 2), PhCF_3_ shows preferential adsorption on graphite due to the favorable electrostatic effect. Both of which might influence the interfacial behaviors and will be discussed later.

### 2.2 Graphite Reversibility and Co‐Intercalation Mechanisms

**Figure 3:**
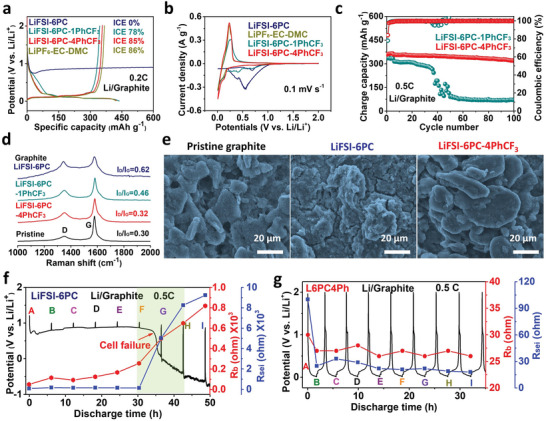
Graphite compatibility and PC co‐intercalation mechanism. a) The initial charge–discharge curves, b) CV results and c) cycling stabilities of Li/graphite cells in different electrolyte (1 C is defined as 360 mAh g^−1^ for graphite). d) Raman results and e) SEM images of graphite cycled in different electrolytes. Discharge curves and interfacial resistances variation of graphite in f) LiFIS‐6PC and g) L6PC4Ph.

The reversibility of graphite is assessed in Li/graphite half cells. A long voltage plateau situated at 0.8 V can be noticed during discharging in LiFSI‐6PC (**Figure** **3**a), attributing to PC co‐intercalation and decomposition. By contrast, graphite cycles reversibly once PhCF_3_ is introduced in LiFSI‐6PC‐1PhCF_3_ (L6PC1Ph), showing inhibited initial cathodic current within 0.9 and 0.3 V in CV results (Figure 3a,b). Moreover, the increased ICEs from 78% (L6PC1Ph) to 85% is obtained after increasing PhCF_3_ content to LiFSI‐6PC‐4PhCF_3_ (L6PC4Ph) (Figure 3a). The enhanced reversibility of graphite upon PhCF_3_ addition is further revealed by CV results with overlapped current peaks (Figure S7, Supporting Information). LiFSI‐6PC electrolyte with varied contents of PhCF_3_ are compared (Figure S8, Supporting Information), showing higher rate capability, ICE and conductivity for L6PC4Ph. The interaction between PhCF_3_ and anion can immobilize FSI^−^ and might leads to higher Li^+^ transference number, which will be beneficial for improving rate capability. Note that the addition of PhCF_3_ at lower Li salts’ concentration (LiFSI‐7PC‐4PhCF_3_ and LiFSI‐10PC‐4PhCF_3_) also inhibits co‐intercalation but suffers from inferior cycling stability compared with L6PC4Ph (Figure S9, Supporting Information). As a result, L6PC4Ph is used as the optimized formulation because of the acceptable ICE (85%) and good reversibility, which is comparable to those of EC‐based electrolytes (1 m LiPF_6_ dissolved in EC and DMC, 1:1 vol%) (Figure 3a and Figure S10, Supporting Information). Moreover, graphite in L6PC4Ph keeps 90% capacity retention after 100 cycles at 0.5 C, much higher than that of L6PC1Ph (20% capacity retention) (Figure 3c). The fast capacity fade in L6PC1Ph is probably caused by the relatively poor SEI due to limited PhCF_3_. Moreover, the cycling stabilities of graphite in EC‐based electrolyte (Figure S10, Supporting Information) and SCE (Figure S11, Supporting Information) are also investigated, showing limited capacity and high voltage polarization for SCE (LiFSI‐3PC) since high viscosity and low conductivity.

To reveal the graphite failure mechanism in LiFSI‐6PC electrolyte, Raman spectra are collected in Figure 3d, showing increased I_D_/I_G_ values after cycling. The significantly decreased G peak intensity for graphite in LiFSI‐6PC indicates the highly disordered structure, which is agreement with the XRD results showing broad peak and elimination of diffraction from (002) lattice plane (Figure S12, Supporting Information). These results indicate the substantially destructed graphite lattice during PC co‐intercalation.^[^ ^35^, ^36^ ^]^ Nevertheless, the structural degradation is ameliorated via introducing PhCF_3_ as denoted by the remained I_D_/I_G_ values in Raman results and distinct diffraction peaks in XRD patterns (Figure 3d and Figure S12, Supporting Information). In addition, the graphite electrode shows fluffy surface and gets disintegrated from current collector after cycling in LiFSI‐6PC, which is different from the smooth surface observed in L6PC4Ph (Figure S12, Supporting Information). The morphological evolutions of graphite are further investigated in SEM images, with thick and heterogeneous by‐products accumulated on graphite surface for LiFSI‐6PC (Figure 3e). In contrast, the graphite is cover uniformly when cycled in L6PC4Ph, implying the superior film‐forming capability in designed electrolyte. Note that the anions‐derived protection in L6PC4Ph is also revealed by the uniform distribution of F and S elements (Figure S13, Supporting Information).

Given the significantly varied surface morphologies, in situ EIS is used to monitor the interfacial resistance during cathodic process in LiFSI‐6PC (Figure 3f and Figure S14, Supporting Information). The cell failure is observed after discharging at 0.5 C for 35 h as denoted by fast voltage decline and sharply increased *R*_b_ (bulk resistance) and *R*_sei_ (SEI resistance), indicating the gradual depletion of electrolyte and uncontrollable SEI growth during PC co‐intercalation. By contrast, the graphite cycles reversibly in L6PC4Ph with gradually decreased *R*_b_ and *R*_sei_ after initial activation process (Figure 3g), demonstrating the formation of competent SEI for kinetically stabilizing graphite and thus ensuring reversible operations. As a result, graphite suffers from both structural destruction and interfacial degradation upon discharging in LiFSI‐6PC electrolyte. The graphite operated in L6PC4Ph, nevertheless, preserves stable lattice and sustains reversible cycling. The improved performance can be ascribed to the anions‐derived protection in L6PC4Ph, which originates from the surfactant effect of PhCF_3_.

### 2.3 Surfactant Effect of PhCF_3_ and SEI Characterizations

**Figure 4:**
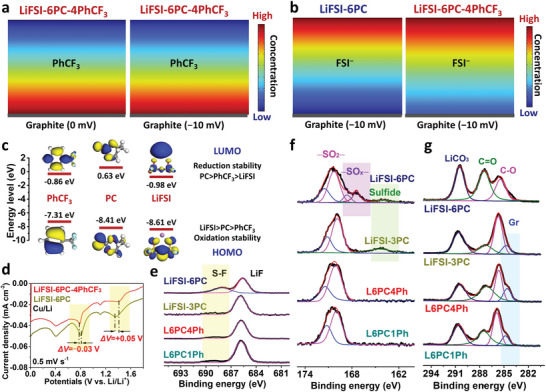
Interfacial simulations and SEI characterizations. a) The interfacial concentration of PhCF_3_ at neutralized and negatively charged graphite. b) The interfacial concentration of FSI^−^ for L6PC and L6PC4Ph at negatively charged graphite. c) The highest occupied molecular orbital (HOMO) and the lowest unoccupied molecular orbital (LUMO) energy levels for different components. d) LSV results of different electrolytes on Cu foil. e) F, f) S, and g) C signals from XPS results of graphite in different electrolyte.

To understand the surfactant effect of PhCF_3_ in PC‐based electrolyte, the interfacial behaviors are studied by theoretical simulations. As shown in **Figure** **4**a, PhCF_3_ accumulates near graphite/electrolyte interface due to the favorable *π*‐*π* stacking between graphite and PhCF_3_ (Figure 2g). Moreover, the distribution of other electrolyte components is visualized based on COMSOL simulations (Figures S15–S17, Supporting Information), showing a gradual decrease of FSI^−^ concentration from bulk electrolyte to interface (Figure 4b), agreeing well with the strong electrostatic repulsion between negatively charged surface and anions.^[^ ^25^, ^27^ ^]^ However, a relatively high concentration of FSI^−^ is obtained for L6PC4Ph near graphite surface (Figure 4b and Figure S18, Supporting Information), validating the benign effect of PhCF_3_ for counteracting electrostatic repulsion, which originates from ion–dipole interaction between PhCF_3_ and FSI^−^. Moreover, the accumulation of PhCF_3_ and FSI^−^ exclude PC from electrode/electrolyte interface (Figure S18, Supporting Information), contributing to shielding effects to protect free PC from detrimental decomposition. As a result, the adsorbed PhCF_3_ (*π*–*π* stacking) facilitate accumulation of FSI^−^ (ion–dipole interactions) and exclusion of PC (steric effects) near graphite/electrolyte interface at low Li salts’ concentration (L6PC4Ph, 1 m), switching from PC co‐intercalation to reversible Li^+^ intercalation, which is similar to the interfacial chemistry of SCEs (Li salts >3 m).

The interfacial chemistry is revealed by theoretical calculations and experiments. LiFSI possesses the lowest energy level of LUMO (Figure 4c), denoting the higher propensity to get electron during SEI formation. However, the strong electrostatic repulsion near graphite surface inhibits anions’ reduction, leading to fluffy and uneven organic‐dominated SEI in L6PC (Figure 3e). As shown in linear sweep voltammetry (LSV) results (Figure 4d), the reduction of FSI^−^ and PC, shift from 1.36 to 1.41 V and 0.8 to 0.77 V, respectively, upon PhCF_3_ addition, resulting from the facilitated anions’ reduction and delayed PC's reduction on Cu foil.^[^ ^37^ ^]^ No detectable PhCF_3_ reduction peak can be observed in LSV results since its higher LUMO than LiFSI. The SEI on graphite is studied by XPS results (Figure S19, Supporting Information). The outweighed LiF is observed for L6PC1Ph and L6PC4Ph (Figure 4e), similar to that of SCE (LiFSI‐3PC), denoting the dominated FSI^−^ decomposition. By contrast, the coexistence of S‐F and LiF for L6PC indicates the plagued transformation from LiFSI to LiF.^[^ ^38^ ^]^ Moreover, the appearance of –SO_x_– and sulfide further reveal the different reduction pathways for FSI^−^ on graphite surface (Figure 4f), leading to unfavorable SEI and cell failure in L6PC. As shown in Figure 4g, intensified LiCO_3_ and C = O are observed for SEI in L6PC, suggesting the aggravated PC decomposition. Furthermore, the graphite in LiFSI‐6PC is covered with thick organic components as denoted by the disappearance of graphite signal in C 1s spectra, which is coincident with SEM images (Figure 3e) and EIS results (Figure S14, Supporting Information). In contrast, the decomposition of FSI^−^ anions is facilitated and PC is inhibited in L6PC4Ph, forming reliable interphase consisting of LiF and Li_2_CO_3_, similar to that of SCE (LiFSI‐3PC), to kinetically stabilize graphite in PC electrolyte.

### 2.4 Adsorption–Attraction–Reduction Mechanism

**Figure 5:**
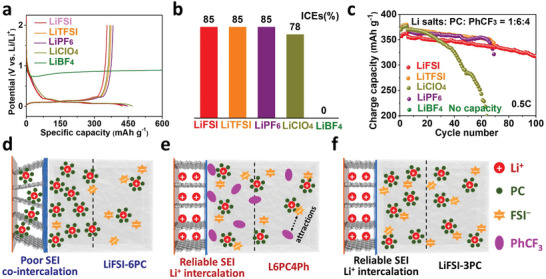
Interfacial mechanism. a) The initial charge–discharge curves, b) ICEs, and c) cycling stabilities of graphite/Li cells in various electrolytes (Li salts: PC: PhCF3 = 1:6:4). d) The schematic illustrations for LiFSI‐6PC (d), L6PC4Ph (c), and SCE (LiFSI‐3PC, >3 m) (c).

To establish the decisive roles of anions’ reduction rather than the defluorination of PhCF_3_ for stabilizing graphite in PC electrolyte, the initial charge‐discharge curves and ICEs of Li/graphite in various electrolytes are investigated, which shows direct correlation between ICEs and Li salts (**Figure** **5**a,b). The Li salts with good film‐forming capability (LiFSI, LiTFSI and LiPF_6_) deliver high ICEs of 85%, while the electrolyte with LiClO_4_ can only sustain a lower ICE of 78%. Note that SEI formed in LiBF_4_‐6PC‐4PhCF_3_ cannot stabilize graphite and demonstrate ceaseless co‐intercalation (Figure 5a), despite similar electrolyte compositions. These results suggest the anion‐relevant behaviors and highlight the decisive roles of anions’ decomposition rather than the defluorination of PhCF_3_ (Figure 5c). Moreover, the defluorination from PhCF_3_ is thermodynamically disfavored as revealed by theoretical calculations (Figure 4c) and LSV results (Figure 4d). However, the surfactant effect from PhCF_3_ should not be neglected since it adsorbs on graphite surface (*π*–*π* stacking), attracts anions (ion–dipole interactions) and excludes PC (steric effects). Thus, electrolytes with LiFSI, LiPF_6_ and LiTFSI achieve reversible cycling of graphite. Nevertheless, graphite in LiClO_4_‐4PC‐4PhCF_3_ featuring ClO_4_^−^ derived SEI suffers from fast capacity fading. Moreover, graphite in LiBF_4_‐6PC‐4PhCF_3_ undergoes endless co‐intercalation and cell failure. Note that LiTFSI suffers from Al corrosion at high voltage and LiPF_6_ is hampered by its sensitivity toward humidity and high temperature, both of which endorse LiFSI as promising candidate for advanced electrolyte.

Given the ion–dipole interactions and specific adsorption of PhCF_3_, an adsorption–attraction–reduction mechanism is proposed near graphite surface. In L6PC, PC coordinates with Li^+^ to form stable solvation structure. It is hard for FSI^−^ anions to approach graphite surface due to electrostatic repulsion, which is stripped off immediately during discharging (Figure 5d). Therefore, side reactions and co‐intercalation proceed in graphite with aggravated decomposition from both PC solvent and FSI^−^ anions.^[^ ^29^, ^39^ ^]^ In L6PC4Ph, the preferentially adsorbed PhCF_3_ attracts FSI^−^ anions without impairing Li^+^ coordination via ion–dipole interactions (Figure 5e), which compensates electrostatic repulsion and induces accumulation of FSI^−^ on graphite/electrolyte interface, leading to anion‐dominated decomposition and inorganic‐rich SEI. The adsorption–attraction–reduction mechanism constructs interfacial high concentration of anions at a relatively low Li salts’ concentration (LiFSI‐6PC, 1.9 m; L6PC4Ph, 1 m) via surfactant effects of PhCF_3_, differing from SCEs that from anions‐derived SEI by increasing overall Li salts’ concentration (above 3 m) (Figure 5f).

### 2.5 High‐Nickel Cathode Compatibility

**Figure 6:**
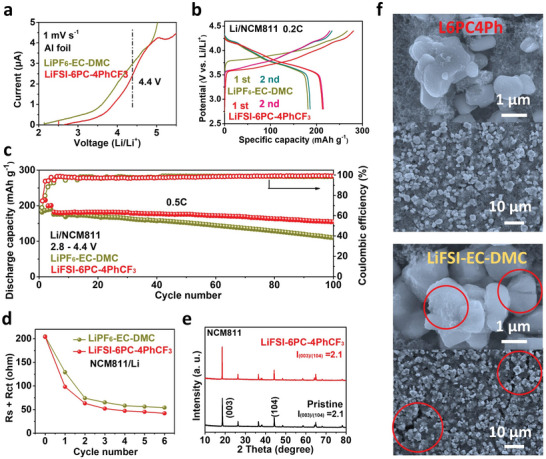
The electrochemical of NCM811. a) LSV results on Al foil. b) Charge‐discharge curves at 0.2 C and c) cycling stabilities at 0.5 C of Li/NCM811 cells in EC‐based electrolyte and L6PC4Ph (1 C is defined as 180 mA g^−1^ for NCM811). d) Interfacial resistance derived from EIS results. e) XRD results of NCM811 in varied electrolytes. f) SEM images of cycled NCM811 in L6PC4Ph and EC‐based electrolyte.

LSV is carried out to reveal the high oxidation stability of L6PC4Ph, showing slightly lower oxidation current at 4.4 V than that of EC‐based electrolyte (**Figure** **6**a). The NCM811 exhibits similar electrochemical behaviors in both L6PC4Ph and EC‐based electrolyte (Figure S20, Supporting Information). As shown in Figure 6b, a higher discharge capacity of 210 mAh g^−1^ and less voltage polarization are observed at 0.2 C for Li/NCM811 cell in L6PC4Ph. Furthermore, the cell preserves a high capacity retention of 90% after 100 cycles at 0.5C within 2.8 to 4.4 V, much better than that of EC‐based electrolyte (Figure 6c). The fast capacity fading in EC‐based electrolyte is probably caused by the electrode‐crosstalk and cathode surface degradation.

As shown in Figure 6d, NCM811 exhibits high initial interfacial resistance in both L6PC4Ph and EC‐based electrolyte. The resistance declines after initial activation, indicating the formation of interphase on cathode.^[^ ^40^ ^]^ However, a higher interfacial resistance can still be observed for NCM811 in EC‐based electrolyte, suggesting a relatively poor surface protection (Figure 6d and Figure S21, Supporting Information). XPS results indicate the organic‐rich CEI on NCM811 cycled in EC‐based electrolyte since the higher contents of C‐O and C = O species (Figure S22, Supporting Information). Moreover, less LiF is obtained as denoted by the F 1s spectra. The organic‐rich CEI is resistive and ineffective for interfacial protection, leading to severe transition metal dissolution (Figure S23, Supporting Information) and possible structural degradation. As a result, the NCM811 cycled in EC‐based electrolyte exhibits rough surface, which is derived from the accumulation of interfacial by‐products. Moreover, some micro‐cracks are observed, indicating the detrimental lattice evolutions in EC‐based electrolyte. In contrast, the XRD patterns show preserved layered lattice of NCM811 after cycling in L6PC4Ph, coincident with the good cycling stability (Figure 6e). Moreover, the perfect surface protection of NCM811 in L6PC4Ph is demonstrated by SEM images with smooth surface (Figure 6f and Figure S24, Supporting Information). Overall, the PC‐based electrolyte (L6PC4Ph) shows higher superiority than that of EC‐based electrolyte for high‐nickel cathode (NCM811) in terms of interfacial protection and bulk structure.

### 2.6 Electrolyte Characterizations and Pouch Cell Performance

**Figure 7:**
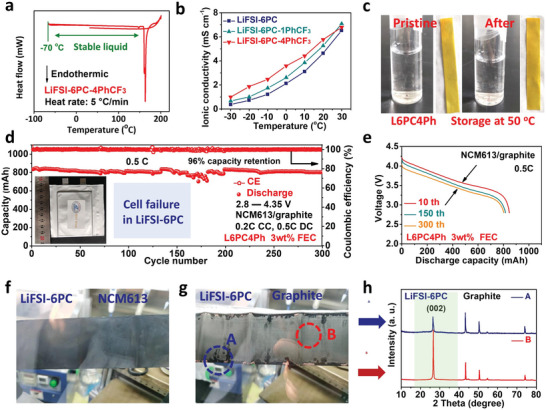
Characterizations of L6PC4Ph and performance of NCM613/graphite pouch cell. a) DSC results and b) conductivities of varied electrolytes. c) The photos of L6PC4Ph at 50 °C after 5 days. d) The cycling performance of NCM613/graphite pouch cell at 0.5 C and e) corresponding charge‐discharge curves. Photos of f) NCM613 electrodes and g) graphite electrodes disassembled from pouch cell in LiFSI‐6PC electrolyte. h) The XRD patterns of graphite on different areas.

The wide temperature adaptability of L6PC4Ph is revealed by DSC results, showing stable liquid range from −70 to 160 °C (**Figure** **7**a). Nevertheless, the EC‐based electrolyte gets frozen at −40 °C (Figure S25, Supporting Information). Moreover, L6PC4Ph shows high conductivity over wide‐temperature range and a decent conductivity of 1 mS cm^−1^ even under −30 °C (Figure 7b). After storage at 50 °C for 5 days (Figure 7c), the L6PC4Ph electrolyte displays similar appearance and pH values, indicating excellent thermal stability.^[^ ^41^ ^]^ Moreover, the wide temperature adaptability of L6PC4Ph is demonstrated in graphite//Li (Figure S26, Supporting Information) and Li_4_Ti_5_O_12_//Li cells (Figure S27, Supporting Information), showing good reversibility even at 60 °C. In addition, improved wettability is obtained for L6PC4Ph with a decreased contact angle of 27° on PP separator, much lower than those of LiFSI‐6PC (66°), EC‐based electrolyte (54°) and L6PC1Ph (46°) (Figure S28, Supporting Information).

The electrochemical performance of NCM613/graphite pouch cell is tested at ambient temperature under practical conditions (NCM613 area capacity: 3.4 mAh cm^−2^, E:C = 3 g Ah^−1^, N:P = 1.1:1). As shown in Figure 7d, the pouch cell delivers a reversible capacity of 830 mAh at 0.5 C and can cycle steadily over 300 cycles with a capacity retention of 96%. The cell keeps stable cycling even when confronted with temperature‐induced fluctuations within 150 to 200 cycles, further demonstrating the reliability of the PC‐based electrolyte (L6PC4Ph). The charge‐discharge curves keep stable and exhibit suppressed voltage decay during long‐term cycling, indicating the practicality of L6PC4Ph for commercial graphite‐based batteries (Figure 7e). Note that a sudden capacity fading is observed after 330 cycles (Figure S29, Supporting Information), which is caused by the depletion of electrolyte and abruptly increased internal resistance. A superior long‐term cycling can be obtained if electrolyte formulation and content is further optimized. In contrast, such pouch cell cannot work in LiFSI‐6PC electrolyte with only endless electrolyte decomposition and distinct gas inflation (Figure S30, Supporting Information). After disassembling the failed pouch cell in LiFSI‐6PC, the NCM613 cathode exhibits a smooth surface with excellent structural integration while the graphite anode shows fluffy areas accumulated mainly on the edge sites (Figure 7f,g), demonstrating the preserved NCM613 cathode but destructed graphite anode. As exhibited in XRD results (Figure 7h), the smooth part of graphite (denoted as B) shows layered graphite lattice with distinct diffraction peaks, but the fluffy part (denoted as A) exhibits significantly decreased (002) intensity, indicating the graphite‐induced failure mechanism in LiFSI‐6PC electrolyte. In contrast, the gas inflation and cell failure are not observed in L6PC4Ph.

## 3 Conclusion

In summary, stable anion‐derived SEI is constructed by introducing PhCF_3_ as a surfactant into PC‐based electrolyte based on the adsorption–attractions–reduction mechanism. PhCF_3_ can attract anions by ion–dipole interactions without impairing the pristine coordination. After the preferential adsorption of PhCF_3_ on graphite surface, more anions are induced toward graphite/electrolyte interface and get decomposed to form anion‐derived SEI. In addition, the electrolyte also exhibits high voltage compatibility and wide‐temperature tolerance. Consequently, the endless PC co‐intercalation and graphite exfoliation are inhibited in designed electrolyte. In NCM613/graphite pouch cells under practical conditions, the anion‐derived SEI preserves a high capacity retention of 96% over 300 cycles at 0.5 C. This work provides a fresh strategy for constructing anion‐derived SEI by adding surfactant into electrolyte to manipulate anions’ behaviors and interfacial chemistry at a relatively low concentration of Li salts.

## Conflict of Interest

The authors declare no conflict of interest.

## Author Contributions

J.X., Z.Z., and M.Q. proposed the concept. M.Q. and Z.Z. designed the experiments. M.Q., Y.W., and R.H. conducted the experiments. X.L. carried out theoretical calculations. All authors contributed to the discussion and writing of the manuscript. J.X. and S. C supervised the project.

## Supporting information

Supporting Information

## Data Availability

The data that support the findings of this study are available from the corresponding author upon reasonable request.
