# Supplementary material for: Revealing Surfactant Effect of Trifluoromethylbenzene in Medium‐Concentrated PC Electrolyte for Advanced Lithium‐Ion Batteries
Source: Adv Sci (Weinh). 2023 Feb 19;10(12):2206648. doi: 10.1002/advs.202206648 (PMC10131810; doi:10.1002/advs.202206648)
Supplement: Supplementary file 1 — Supporting Information [file ADVS-10-2206648-s001.pdf]

ADVANCED SCIENCE
Open Access

Supporting Information

for *Adv. Sci.*, DOI 10.1002/advs.202206648

Revealing Surfactant Effect of Trifluoromethylbenzene in Medium-Concentrated PC Electrolyte for Advanced Lithium-Ion Batteries

Mingsheng Qin, Ziqi Zeng, Xiaowei Liu, Yuanke Wu, Renjie He, Wei Zhong, Shijie Cheng and Jia Xie**

Supporting Information

Revealing Surfactant Effect of Trifluoromethylbenzene in Medium-concentrated PC Electrolyte for Advanced Lithium-ion batteries

*Mingsheng Qin, Ziqi Zeng**, *Xiaowei Liu, Yuanke Wu, Renjie He, Wei Zhong, Shijie Cheng and Jia Xie**

Experimental Section

Electrolyte and electrode preparation

Lithium bis(fluorosulfonyl)imide (LiFSI, 99.5%), $LiClO_4$ (99.5%,), $LiFP_6$ (99.5%), LiTFSI (99.5%), $LiBF_4$ (99.5%), dimethyl Carbonate (DMC, 99.9%), propylene carbonate (PC) and ethylene carbonate (EC) were purchased from Dodo Chem (Suzhou, Jiangsu Province, China). Trifluorotoluene ($PhCF_3$, 99%) was provided by Aladdin. Lithium chips (14 mm diameter, 400 μm thickness) were purchased from China Energy Lithium Corp. Carboxymethylcellulose (CMC) binder was purchased from Aladdin. Separators (Celgard 2400) was obtained from China Energy Lithium Co., Ltd. The electrolyte was prepared by blending different components in glove box based on molar ratio. For example, the LiFSI-6PC-4$PhCF_3$ was prepared by dissolving 1 molar LiFSI in 6 molar PC solvents, following by adding 4 molar $PhCF_3$ into above solvent to get transparent electrolyte. The EC-based electrolyte was prepared by dissolving 1 M $LiPF_6$ in mixed EC and DMC (1:1 vol%) solvent. For electrode preparation, the graphite slurry was prepared by uniformly mixing the graphite powders, Super P, and CMC at mass ratio of 8: 1: 1. The resulting slurry was casted on Cu foil and dried for 24 h at 80 °C to get designed anode. The mass loading for graphite is about 3~4 mg cm^{-2}. The cathode was prepared by mixing NCM811, Super P and PVDF at mass ratio of 8: 1: 1. The slurry was casted on carbon-coated Al and dried for 24 h at 100 °C. The mass loading for NCM811 is 2.5~4 mg cm^{-2} in coin cells. The NCM613/graphite pouch cell was assembled in practical conditions (NCM613 area capacity: 3.4 mAh cm^{-2}, electrolyte: 3 g Ah^{-1}, N:P=1.1:1). Moreover, 3wt% FEC was added into electrolyte only for assessing the cycling stability of pouch cell. Note that 3wt% FEC was added to stabilize NCM lattice and inhibit possible electrode crosstalk in pouch cells.

Materials characterizations

The micromorphology of graphite and NCM811 electrodes were characterized by scanning electron microscope SEM (Nova NanoSEM 450). The XPS was carried out on an AXIS-ULTRADLD-600W. Raman spectra were tested by LabRAM HR800 (HORIBA Jobin Yvon IBH Ltd.) with 785 nm laser for electrolyte and 532 nm for electrodes. NMR results were carried out with Ascend 600 MHZ (Bruker. Co., Ltd.). X–ray diffraction (XRD) patterns of graphite and NCM811 electrodes were collected by X-ray Powder diffractometer (Rigaku MiniFlex600).

Electrochemical measurements

CR2032 coin cells were assembled for Li/graphite and Li/NCM811 cells in an Argon-filled

glovebox with H_2O and O_2 contents below 0.1 ppm. Different electrolytes were injected in coin cells for assessing the electrochemical performance. All coin cells (CR2032) were tested on Neware battery test system (CT-4008Tn-5V10mA-164) at room temperature. Cyclic voltammetry (CV) curves of the cells were tested by CHI 600D at various scan rates. The in-situ/ex-situ Electrochemical impedance spectroscopy (EIS) measurements were measured by a Bio-Logic SP-300.The NCM613/graphite pouch cell was tested by charging at 0.2C and discharging at 0.5C at ambient environment. A constant voltage charging at 4.35 V is applied for 30 min at the end of charging process for each cycle.

Theoretical calculations

The calculations presented here were performed using periodic, spin-polarized DFT as implemented in the Cambridge Sequential Total Energy Package (CASTEP). The electron−ion interactions were described by the Generalized Gradient Approximation (GGA) method proposed by Clarke F.H. The PBE functional was used as an exchange−correlation functional approximation and a plane wave basis set with an energy cutoff of 517 eV was applied. The 1 × 1 × 1 Monkhorst−Pack type of k-point sampling was used for optimization. As for the calculation results of HOMO/LUMO and Electrostatic surface distribution potential, the DMOL3 package has been used, while the functional adopted was same as the former CASTEP package. COMSOL Multiphysics software was used to simulate ionic distribution within electrolyte. The simulation was based on finite element method. The electric field and mass transfer field were calculated by coupling the current and mass transfer in the MEMS module.

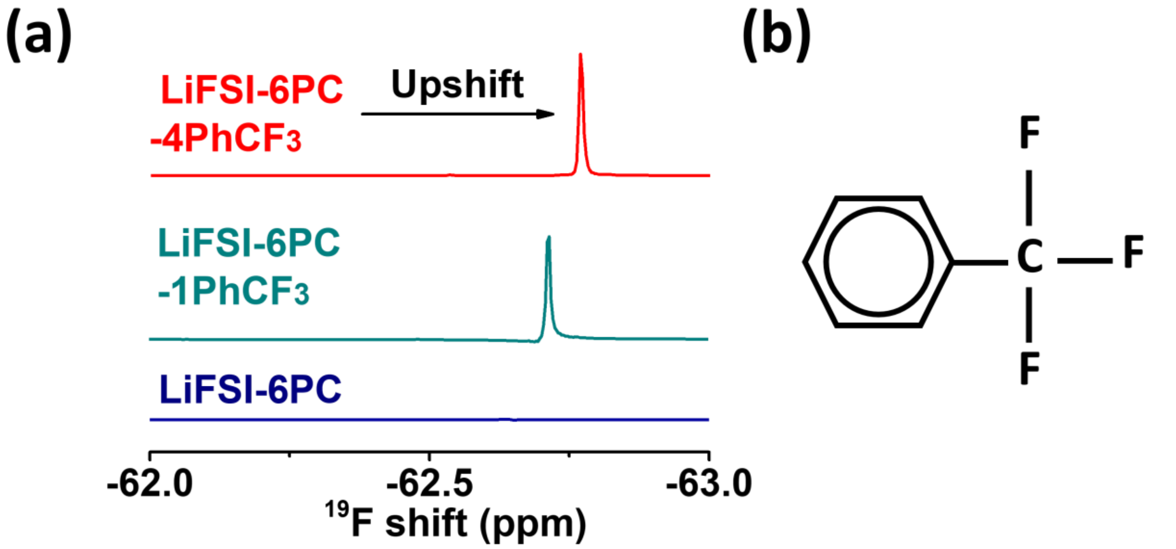

Figure S1. (a) NMR results and (b) molecular diagram of $PhCF_3$. The upshift of ^{19}F for $PhCF_3$ indicates interaction between $PhCF_3$ and anion.

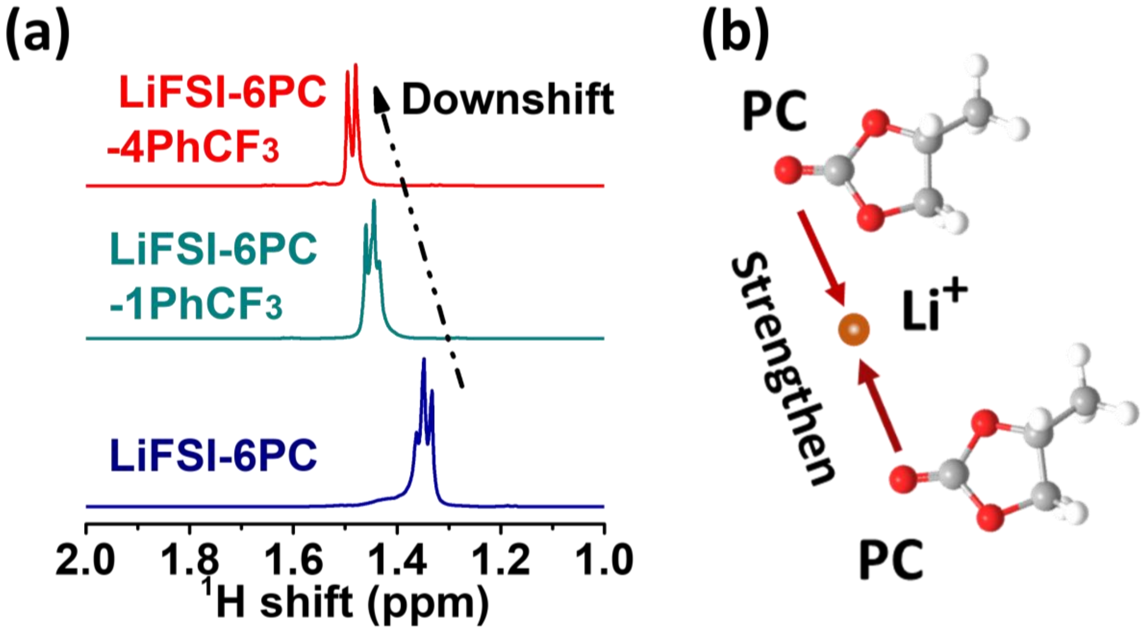

Figure S2. (a) NMR results for PC in different electrolytes and (b) schematic for the strengthened interactions between Li^+ and PC upon $PhCF_3$ addition. $PhCF_3$ immobilizes FSI^- anion and thus facilitate dissolution of Li salts in PC solvent.

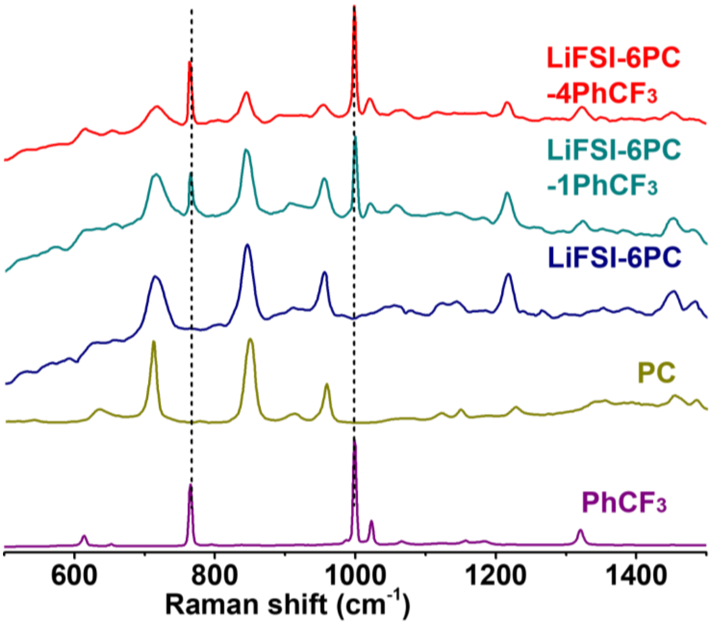

Figure S3. Raman results of different electrolytes.

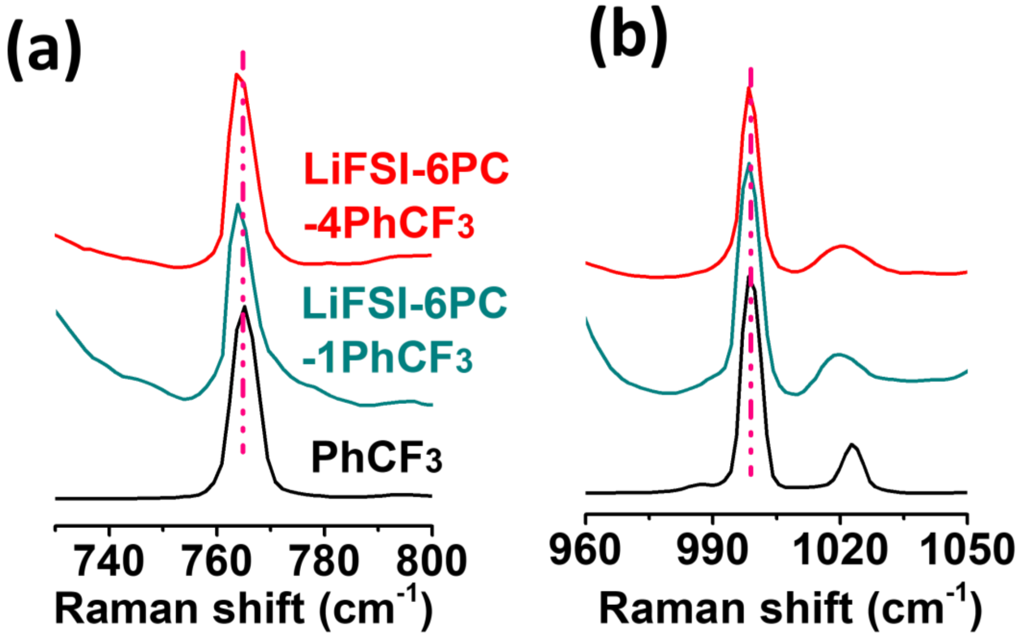

Figure S4. Raman results of $PhCF_3$ in different solvents.

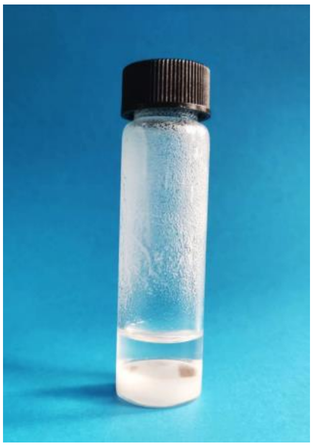

Figure S5. Limited dissolution of LiFSI in $PhCF_3$.

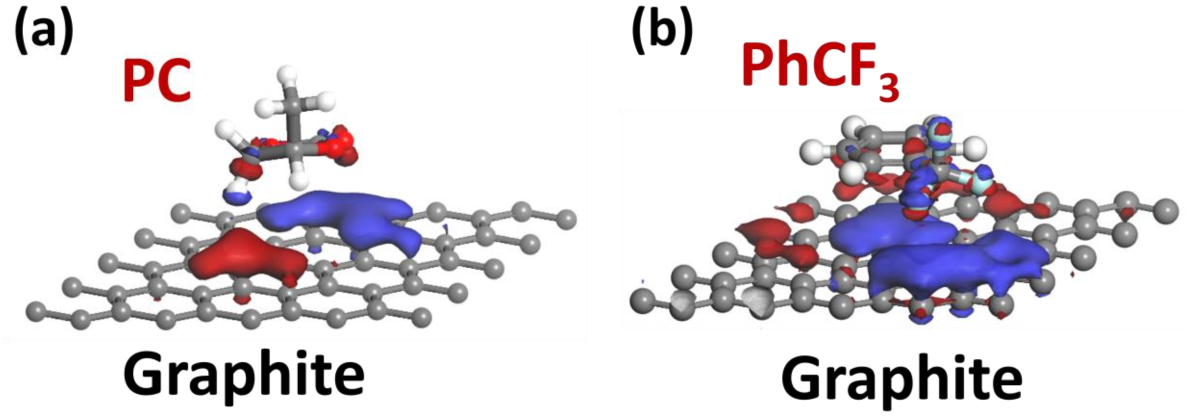

Figure S6. The charge density difference diagram for (a) absorbed PC and (b) $PhCF_3$ on graphite surface. (The blue and red isosurfaces represent charge depletion and charge accumulation, respectively. The isosurface level is 0.0015 au.)

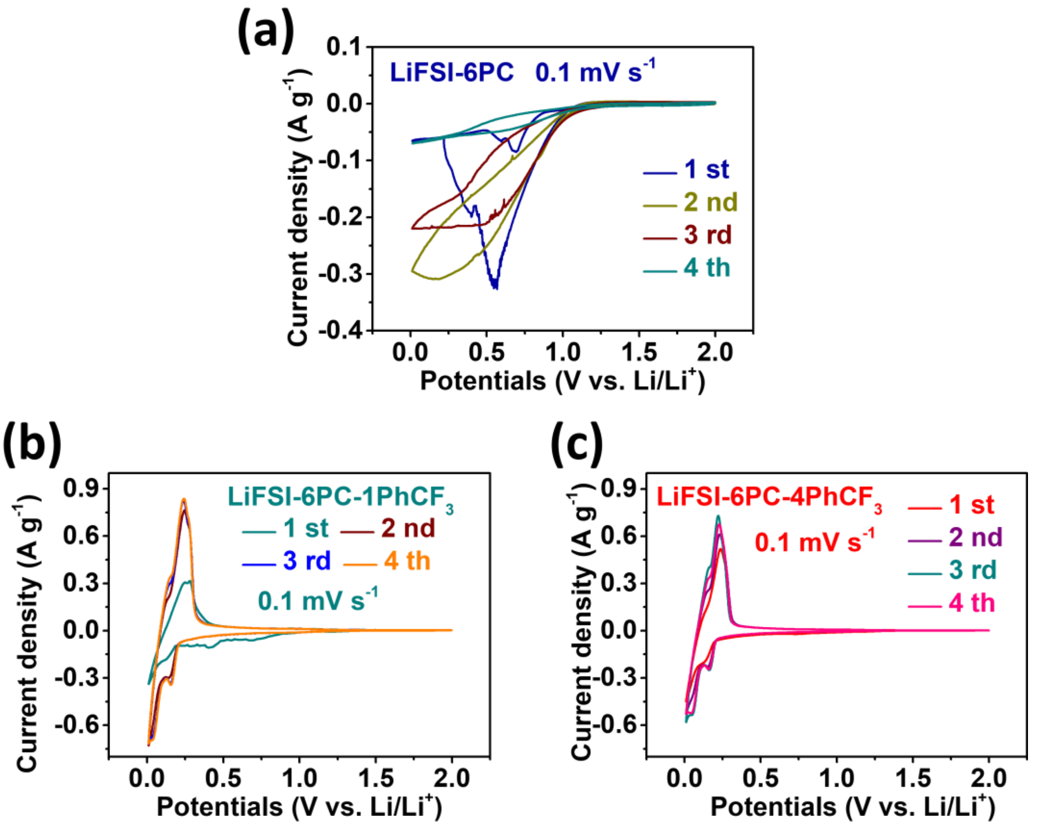

Figure S7. CV curves of graphite in (a) LiFSI-6PC, (b) LiFSI-6PC-1PhCF3 and (c) LiFSI-6PC-4PhCF3 (L6PC4Ph) at 0.1 mV s-1.

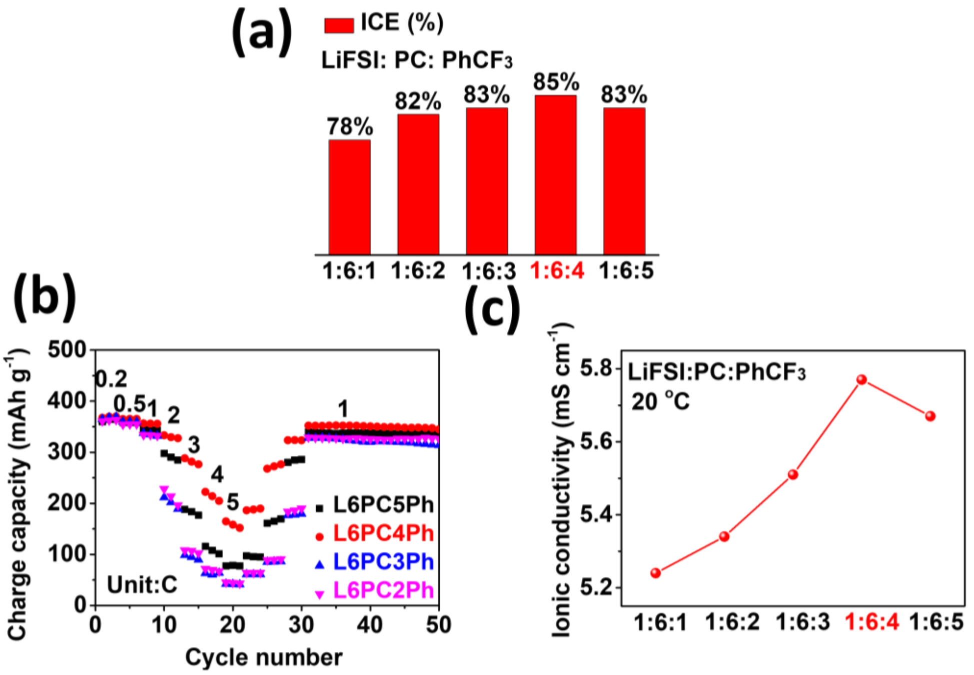

Figure S8. (a) ICEs, (b) rate capabilities of graphite//Li cells and (c) conductivities of LiFSI-6PC with varied contents of $PhCF_3$. The decomposition of $PhCF_3$ is positively correlated with its content. At low content, the decomposition of PC is only partially inhibited as denoted by

the inferior ICEs. As for L6PC4Ph, the interfacial incompatibility of PC and graphite is substantially ameliorated. However, excessive $PhCF_3$ will contribute to inferior interfacial resistance since aggravated decomposition of $PhCF_3$.

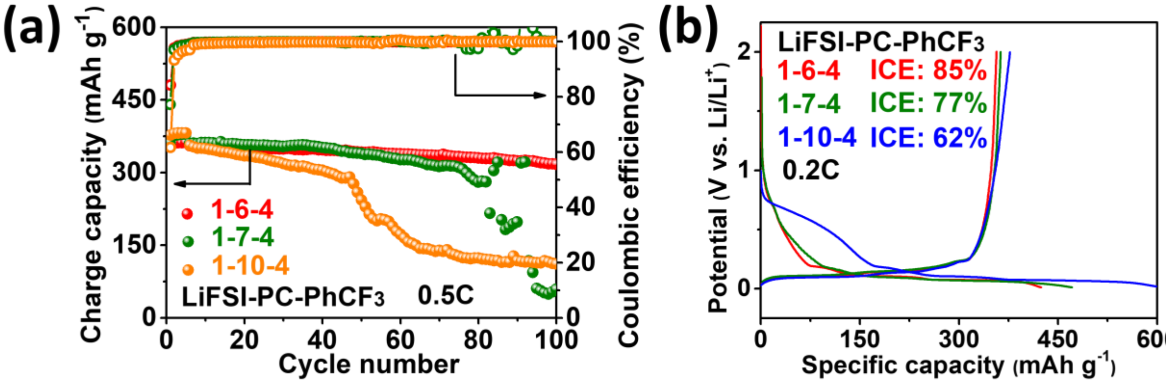

Figure S9. (a) Cycling stabilities and (b) charge-discharge curves of Li/graphite cells in LiFSI-6PC-4PhCF$_3$, LiFSI-7PC-4PhCF$_3$ and LiFSI-10PC-4PhCF$_3$.

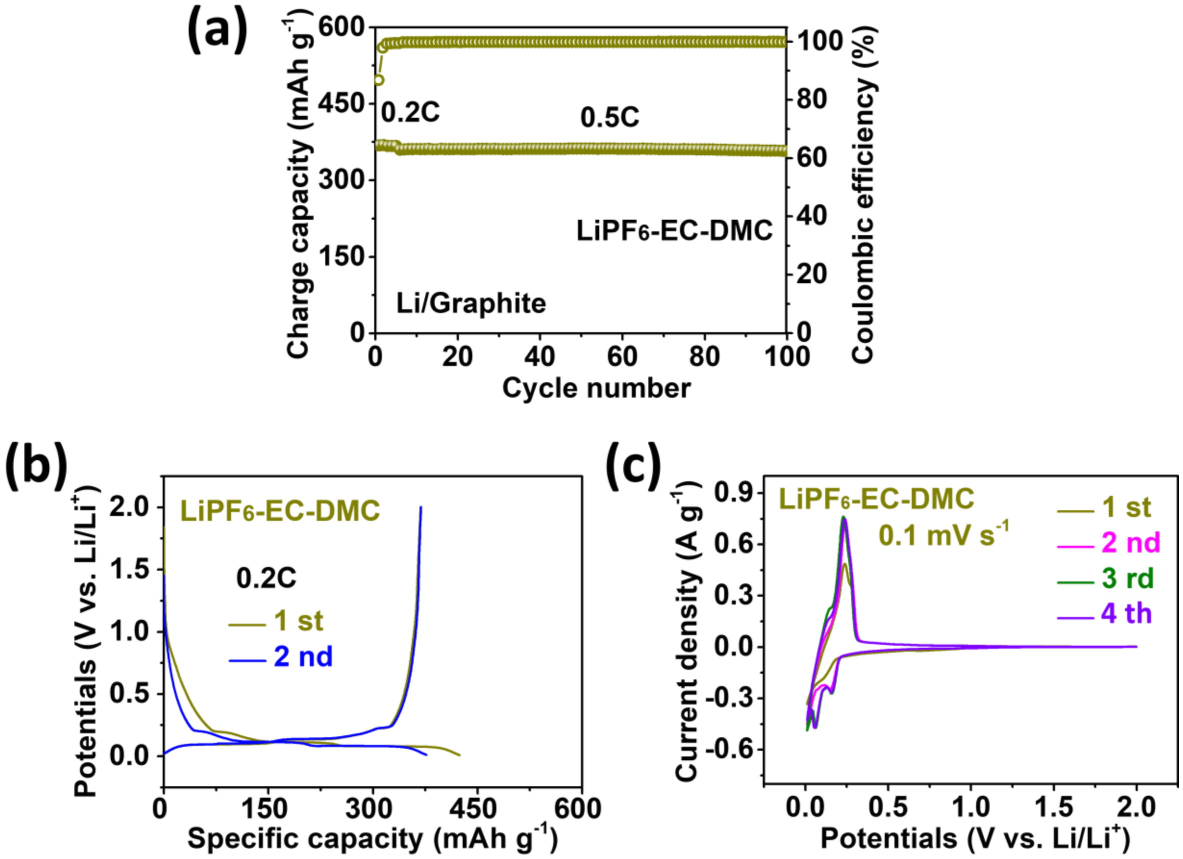

Figure S10. The electrochemical performance of graphite in EC-based electrolyte: (a) cycling stability at 0.5C, (b) charge-discharge curves and (C) CV results.

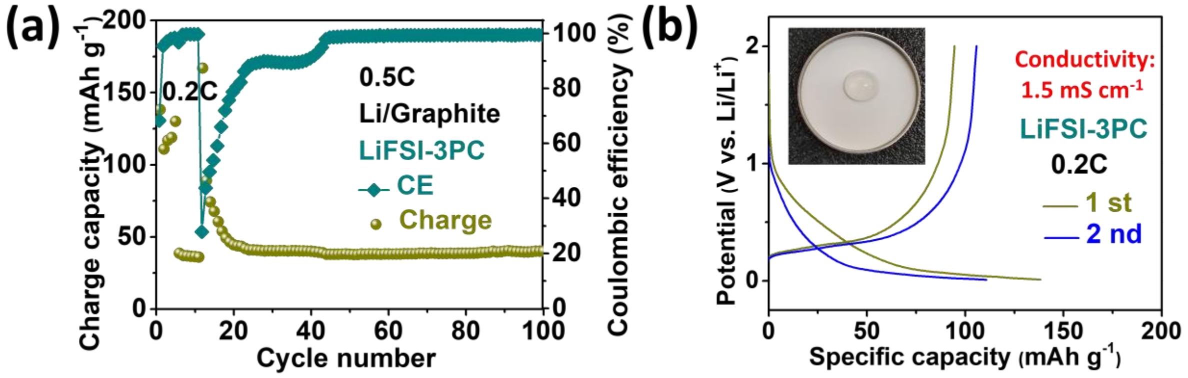

Figure S11. (a) Cycling stability and (b) charge-discharge curves of Li/graphite cells in LiFSI-3PC electrolyte. Insert shows the picture of LiFSI-3PC on PP separator.

The concentrated electrolyte (LiFSI-3PC) enables anions-derived SEI and reversibility for Li/graphite cells since the involvement of anions into solvation shell. Increasing concentration of Li salts changes electrochemical behaviors from solvent co-intercalation (LiFSI-6PC) to reversible cycling (LiFSI-3PC) at the expense of high viscosity and polarization. Moreover, the increased cost also hinders its applications.

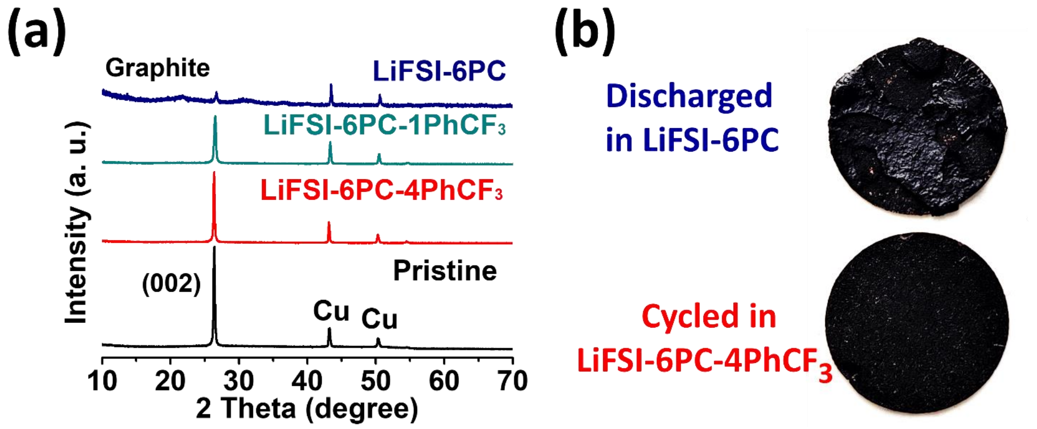

Figure S12. (a) XRD results of graphite cycled in different electrolytes and (b) graphite electrodes disassembled from different electrolytes.

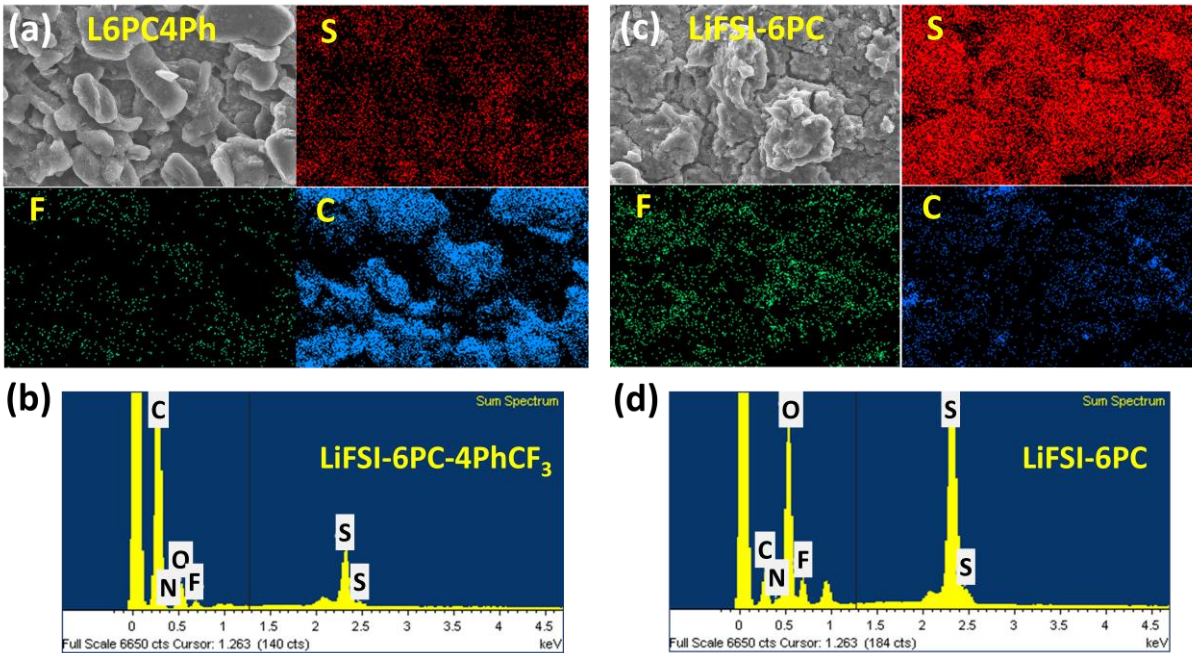

Figure S13. (a) Elemental distribution and (b) contents of cycled graphite in L6PC4Ph. (c) Elemental distribution and (d) contents of discharged graphite (50 h) in LiFSI-6PC. The graphite cycled in LiFSI-6PC was covered by thick by-products with micro-cracks, indicating the aggravated PC and anions decomposition. The poor SEI formed in LiFSI-6PC was failed to protect graphite from further PC co-intercalation.

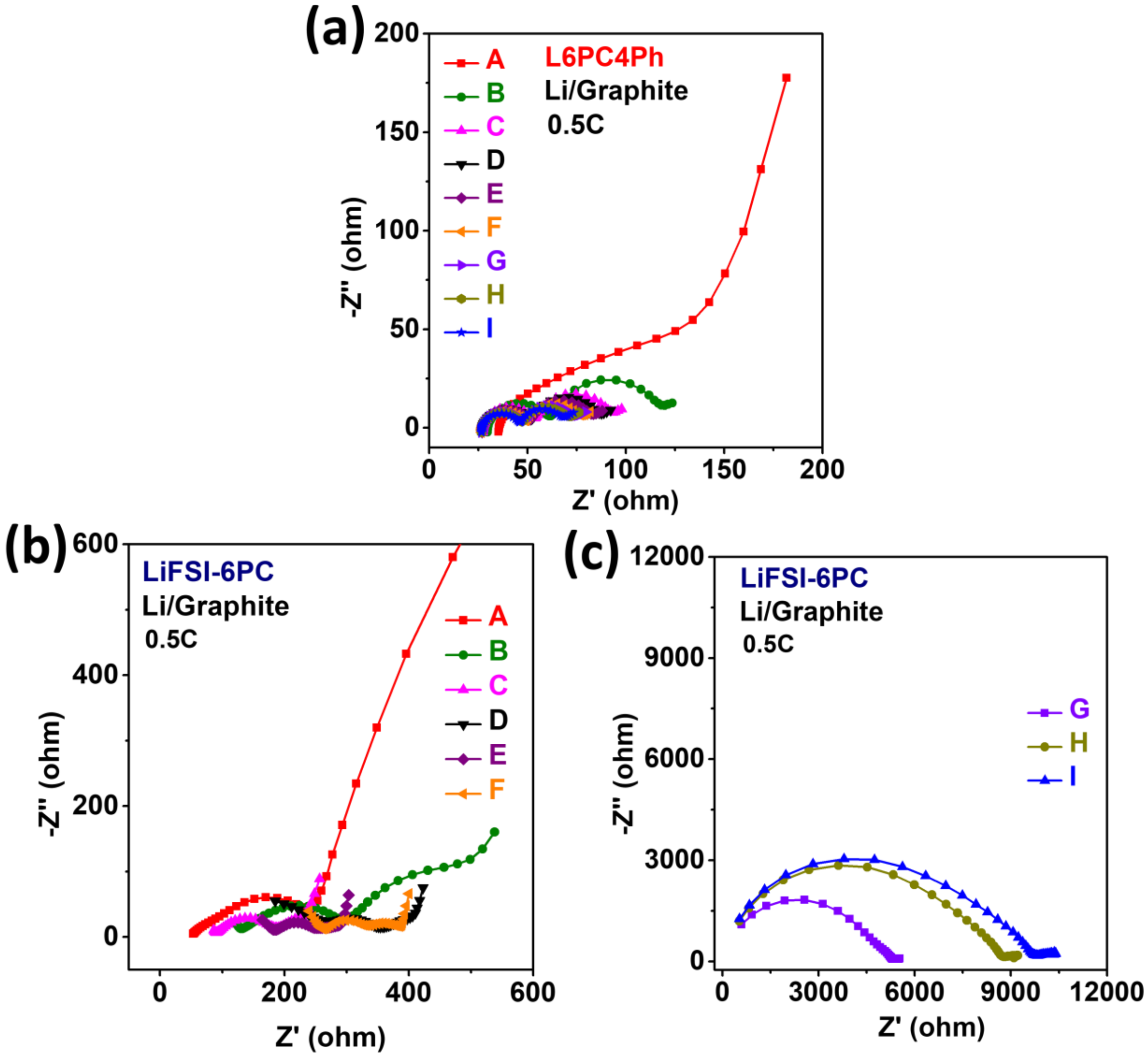

Figure S14. (a) EIS results of graphite in L6PC4Ph. (b), (c) EIS results of graphite after discharging in LiFSI-6PC for varied periods.

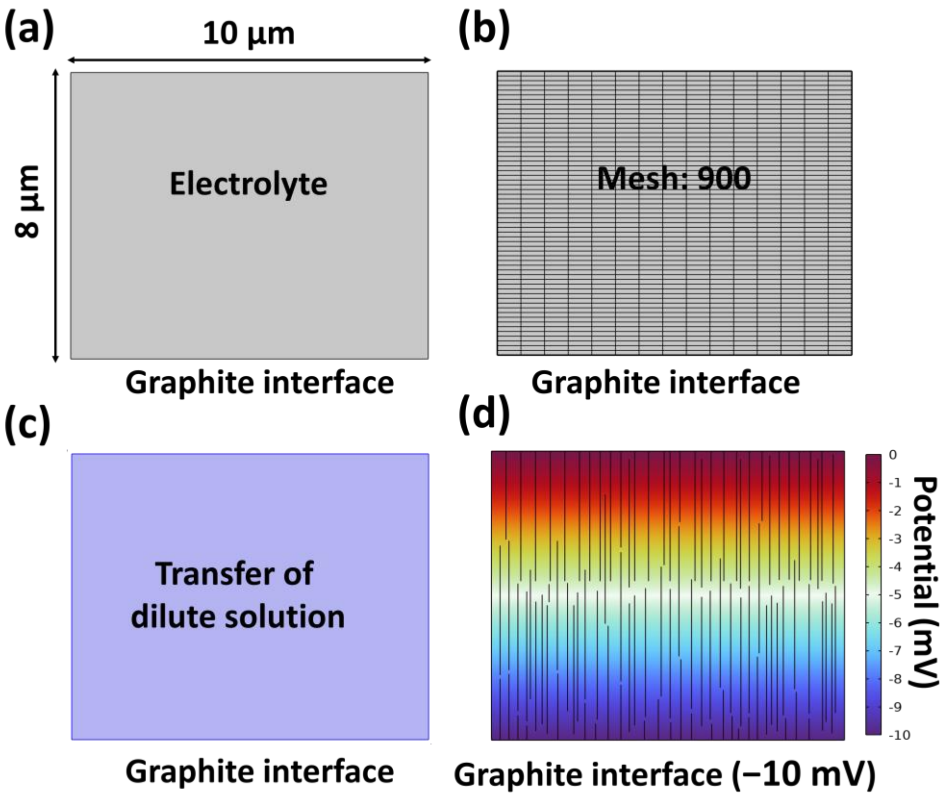

Figure S15. Schematic illustration of the electrolyte model for simulations. (a) The geometry of model with the size of electrolyte solution set to 8 μm×10 μm. (b) The electrolyte region is divided into 900 free mesh under an adaptive grid. (c) The simulations is based on transfer of dilute solution. (d) The top is set to be bulk electrolyte and the bottom is deemed as graphite surface. The electrostatic potential on the bottom boundary is fixed to 0 mV (pristine stage) or −10 mV (discharging).

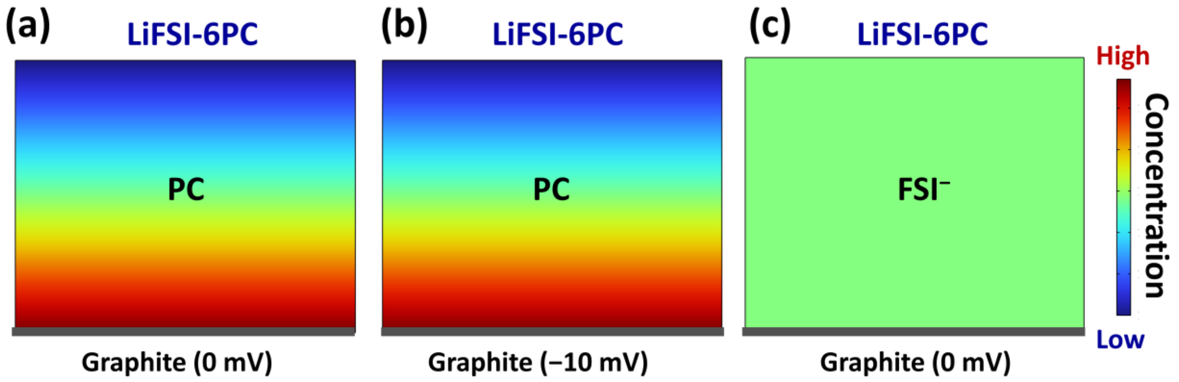

Figure S16. Simulation results of LiFSI-6PC. The distribution of PC (a) at pristine stage and (b) during discharging. (c) The uniformly distributed FSI^{-} at pristine stage. More PC accumulates on graphite surface since their low adsorption energy of −5.34 eV on graphite surface.

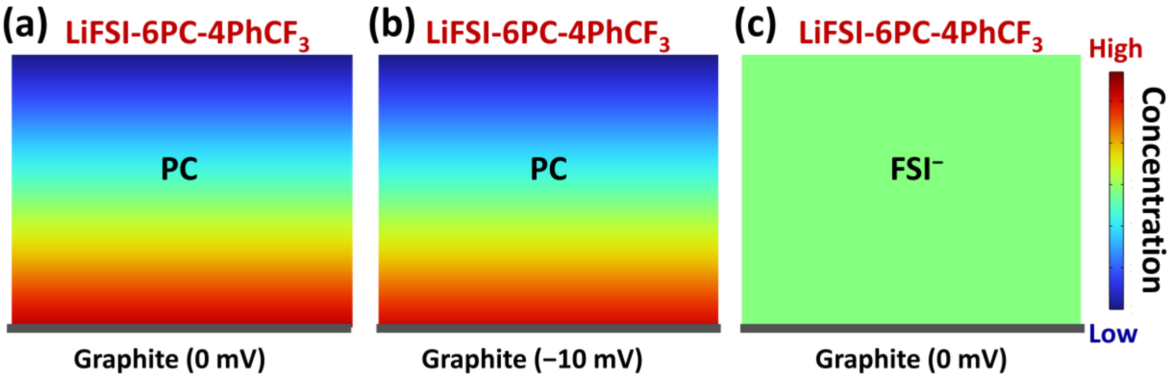

Figure S17. Simulation results of LiFSI-6PC-4PhCF3. The distribution of PC (a) at pristine stage and (b) during discharging. (c) The uniformly distributed FSI^{-} at pristine stage.

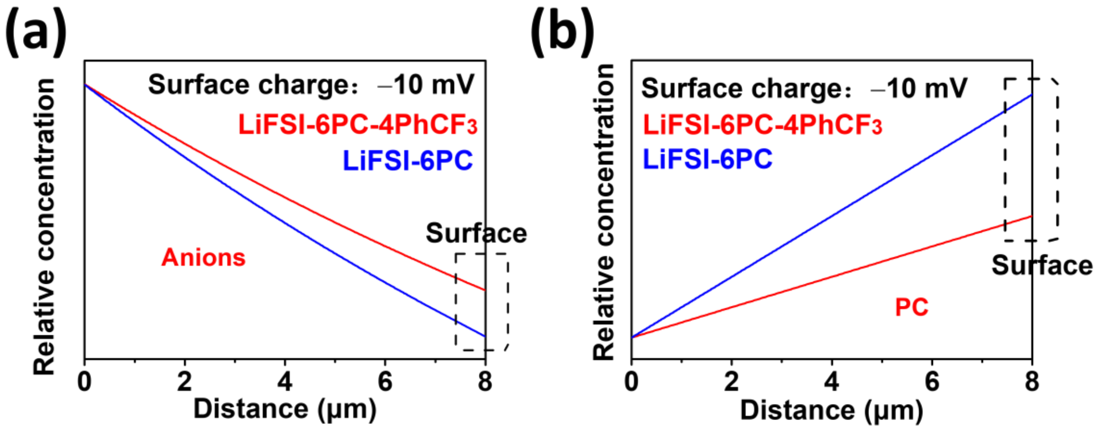

Figure S18. The interfacial concentration of (a) FSI^{-} and (b) PC for L6PC4Ph and LiFSI-6PC as a function of distance. Higher concentration of FSI^{-} and lower concentration of PC are observed for L6PC4Ph near graphite surface.

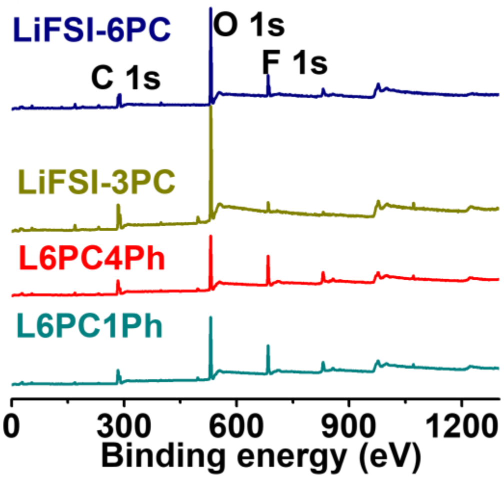

Fgure S19. The XPS results of graphite in different electrolytes.

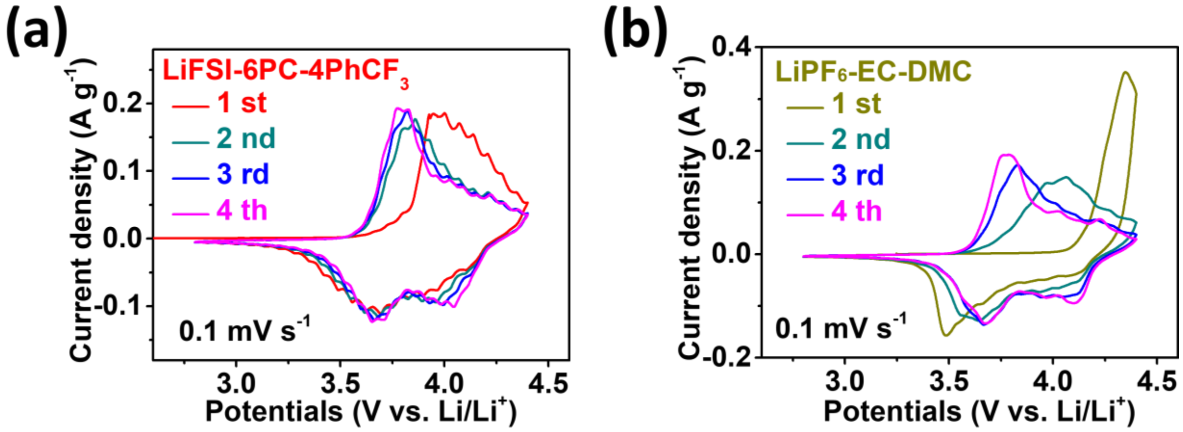

Figure S20. The CV curves of Li/NCM811 cells in (a) L6PC4Ph and (b) EC-based electrolyte. The slight fluctuations of temperature may contribute to unsmooth curves of NCM811 in L6PC4Ph.

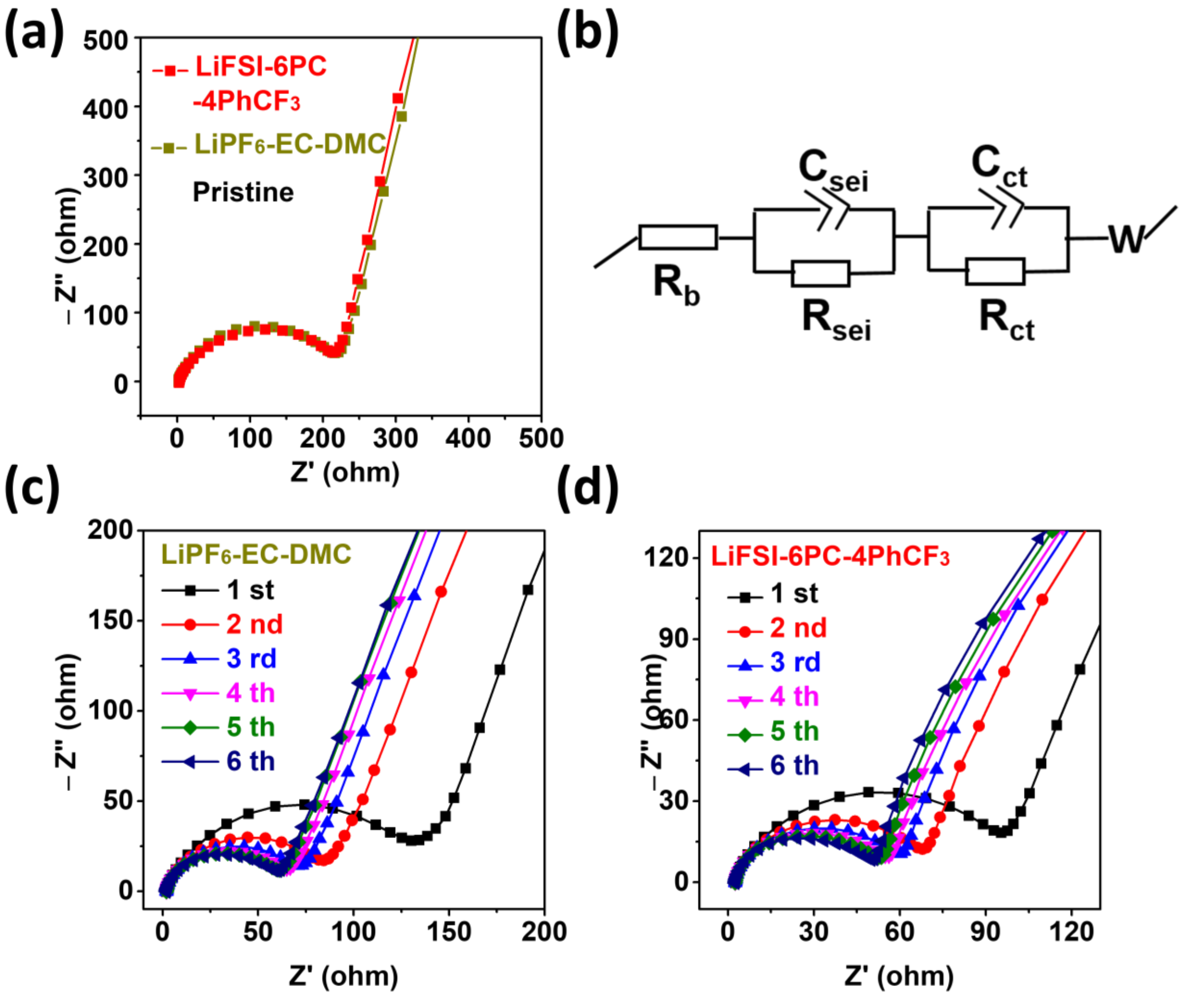

Figure S21. EIS results of (a) pristine NCM811 in L6PC4Ph and EC-based electrolyte. (b) The fitting circuit for EIS results. EIS results of Li/NCM811 in (c) EC-based electrolyte and (d) L6PC4Ph.

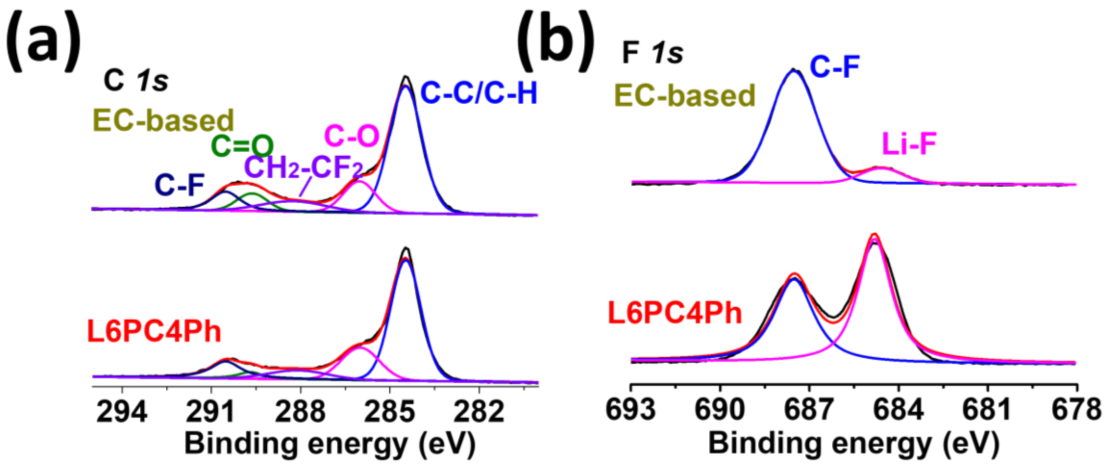

Figure S22. The XPS results of NCM811 cycled in varied electrolytes. (a) C 1s spectra and (b) F 1s spectra.

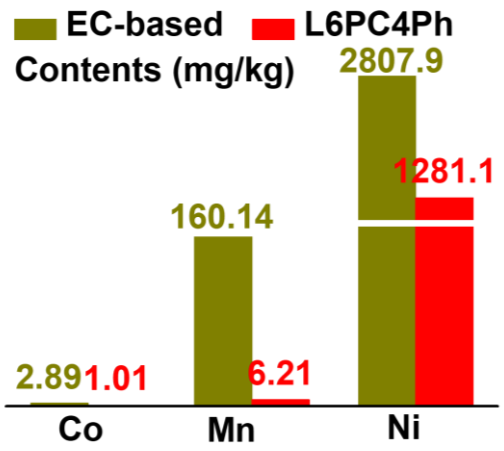

Figure S23. ICP results of PP separator after cycling.

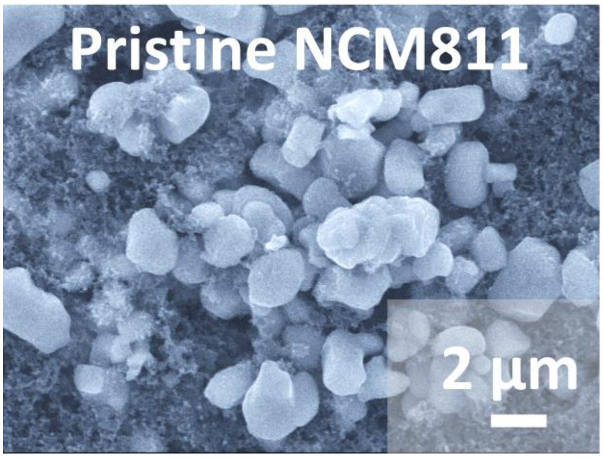

Figure S24. SEM image of pristine NCM811 particles.

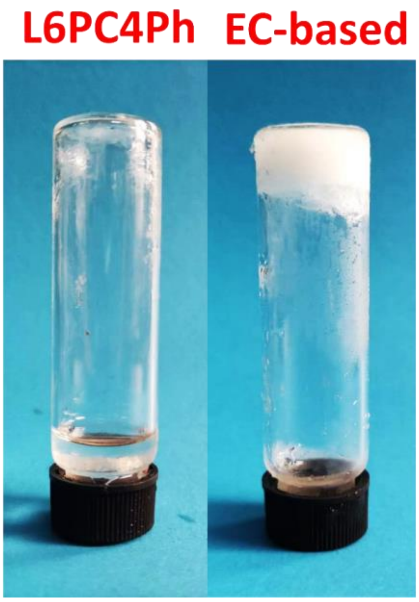

Figure S25. Photos of electrolytes in −40 °C for 2 days.

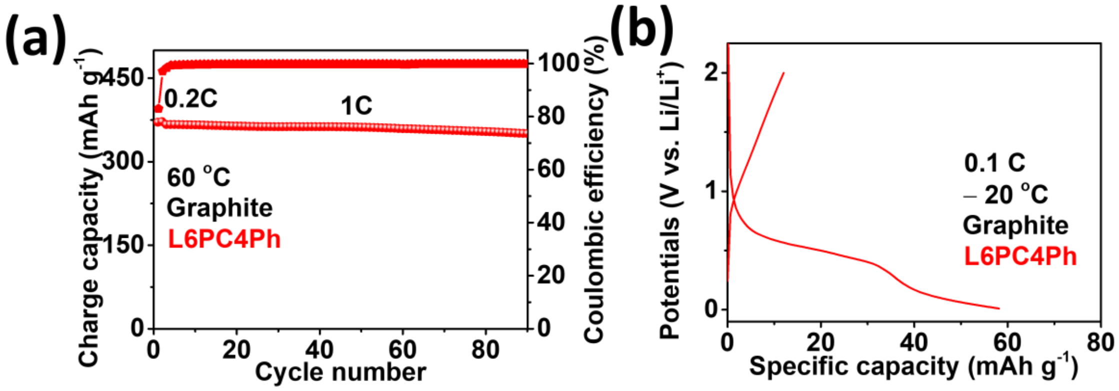

Figure S26. (a) Cycling stability and (b) charge-discharge curves of graphite//Li cells in L6PC4Ph at 60 and -20 °C, respectively.

Graphite//Li cell cycle steadily at 60 °C with a high capacity of 360 mAh g^{-1}, indicating the decent high-temperature stability of L6PC4Ph. Nevertheless, graphite suffers from high polarization and reaches cut-off voltage prematurely at −20 °C, resulted from the sluggish solid-state diffusion at cold condition.

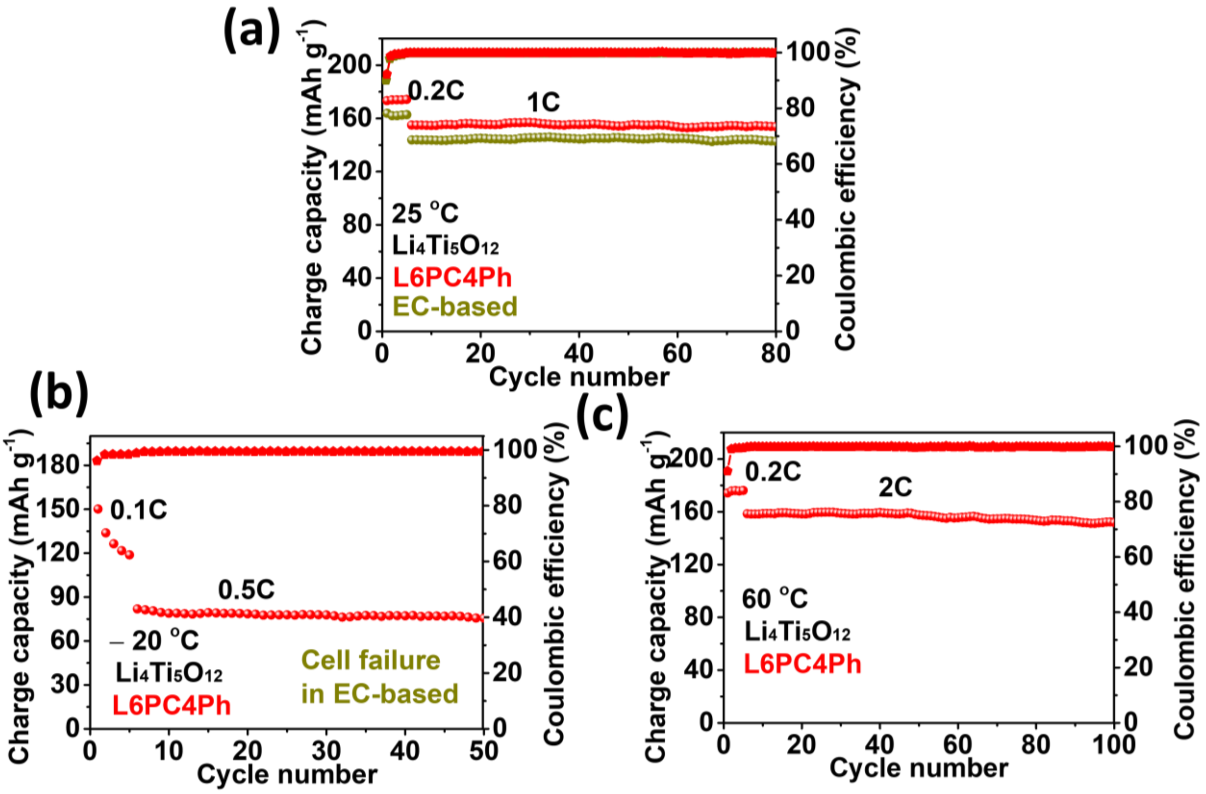

Figure S27. The cycling stabilities of $Li_4Ti_5O_{12}$//Li cells at (a) 25, (b) −20 and (c) 60 °C. $Li_4Ti_5O_{12}$//Li cell can cycle reversibly in L6PC4Ph but exhibits cell failure in EC-based electrolyte at −20 °C. We believe better performance can be obtained if proper surface coating or electrolyte optimization is adopted.

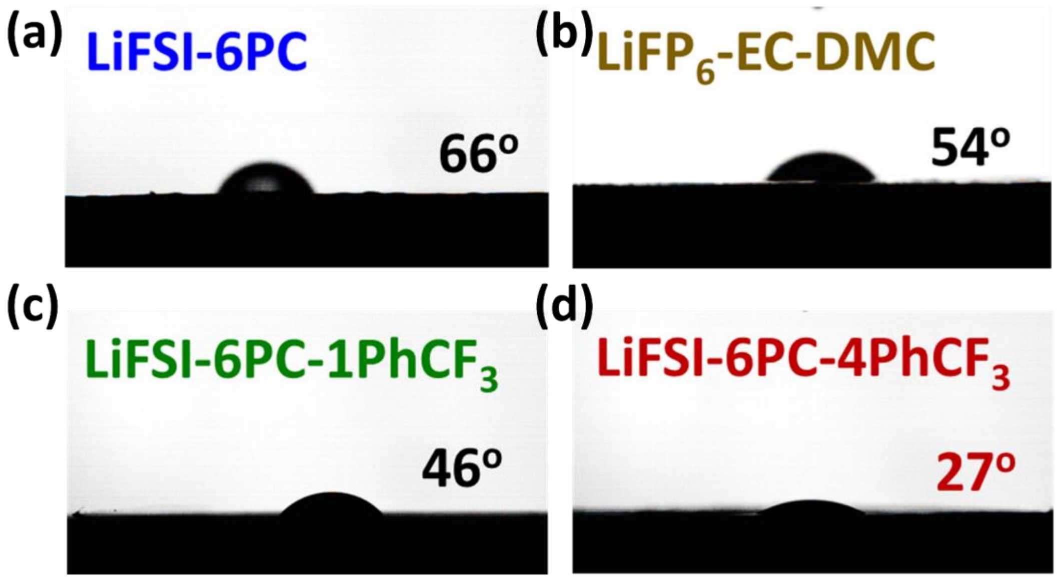

Figure S28. The wettability results of (a) LiFSI-6PC, (b) EC-based electrolyte, (c) LiFSI-6PC-4PhCF$_3$ and (d) L6PC4Ph on PP separators.

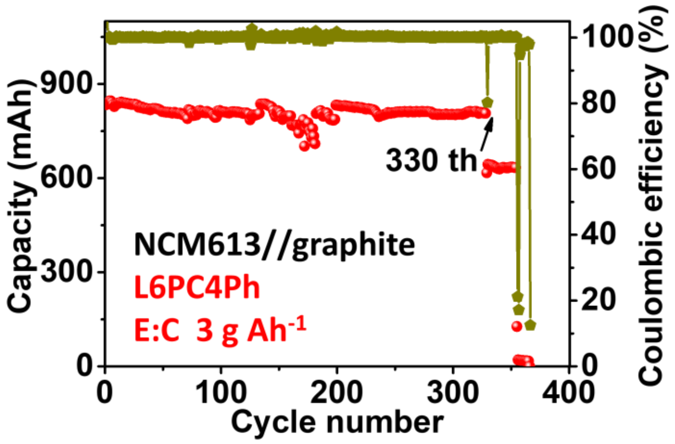

Figure S29. The long-term cycling of NCM613//graphite pouch cell in L6PC4Ph. An abrupt capacity decay is observed due to depletion of electrolyte.

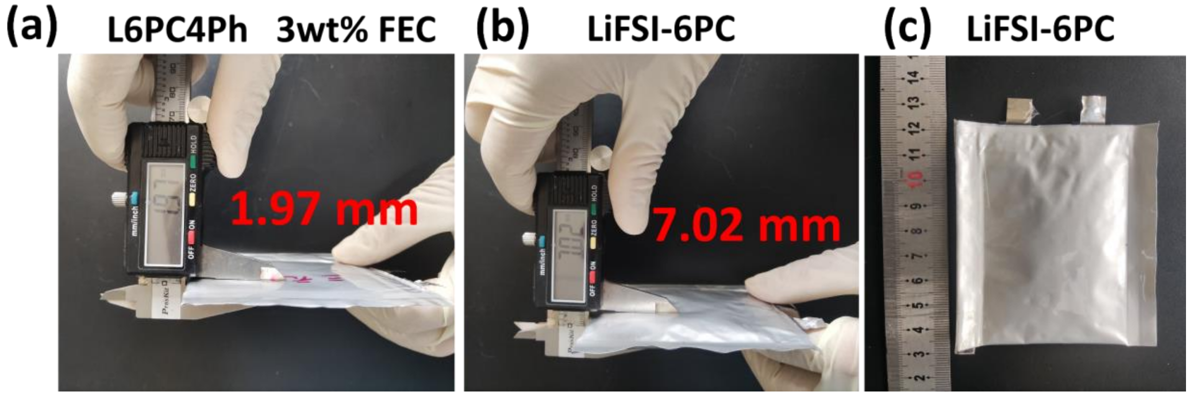

Figure S30. The photos of cycled NCM613/graphite pouch cell in (a) L6PC4Ph and (b) LiFSI-6PC. (c) The inflated pouch cell after charging in LiFSI-6PC.
